# AntibiogramDSM: a combined local antibiogram and educational intervention

**DOI:** 10.1017/ash.2023.450

**Published:** 2023-10-23

**Authors:** Andrew R. Miesner, Benjamin Williamson, Amanda M. Bushman

**Affiliations:** 1 Department of Pharmacy Practice, Drake University College of Pharmacy & Health Sciences, Des Moines, IA, USA; 2 Department of Public Heath, Des Moines University, Des Moines, IA, USA; 3 Department of Pharmacy, UnityPoint Health – Des Moines, Des Moines, IA, USA

## Abstract

**Objective:**

To describe the development of a combined local antibiogram and assess its utility in an educational intervention.

**Design:**

Retrospective analysis of a combined, multi-healthcare system antibiogram with an educational intervention and pre-post analysis.

**Setting:**

Creation of the combined antibiogram included all health systems in Des Moines, Iowa. The educational intervention was delivered live via webinar and remained available on demand for one year.

**Participants:**

The combined antibiogram participants included four health systems representing eight hospitals. The educational intervention included 45 healthcare providers (15 live, 30 on demand) who elected to participate.

**Methods:**

Yearly antibiograms were collected from four health systems for 2017 and 2018 and from three health systems for 2019 and 2020. Each was aggregated into a single antibiogram, posted online, and analyzed retrospectively. In 2021, an educational intervention took place, which included pre-educational assessments, a one-hour presentation on local resistance rates and impact on common infections, and post-education assessments. The educational session was available online for one year. Correct responses before and after education were compared using NcNemar’s test.

**Results:**

Over 4 yr, 123,168 isolates were included in the antibiogram, representing 57 species and 46 tested antibiotics. Before education, prediction of local resistance rates for *E. coli* and *S. pneumoniae* was poor. After the education session, there was improvement in the proportion of correct responses to case-based questions: pneumonia (31.8% vs 58.8%, *P* = 0.022), UTI (47.7% vs 85.3%, *P* < 0.001), sinusitis (75% vs 91.2%, *P* = 0.109), and diverticulitis (43.2% vs 88.2%, *P* = 0.002).

**Conclusions:**

A combined local antibiogram was useful in supporting an outpatient education program.

## Introduction

As antibiotic resistance continues to develop worldwide, it is important to maintain surveillance programs that can identify changes in resistance patterns at a local level and assist prescribers in selecting empiric therapies. One such surveillance tool is the antibiogram, which provides a periodic cumulative report of susceptibility of organisms cultured from an institution’s patient population versus common antibiotics.^1^ Antibiograms are one item highlighted by the Centers for Disease Control and Prevention Core Elements of Hospital Antibiotic Stewardship Programs as a reporting mechanism that supports optimal antibiotic use.^2^ The Infectious Disease Society of America and Society for Healthcare Epidemiology of America guidelines for implementing an antibiotic stewardship program also recommend the use of antibiograms to support empiric treatment guideline development.^3^ They provide a weak recommendation based on limited evidence for antibiograms stratified by hospital location or population, but many smaller institutions may not have adequate bacterial isolate counts to do so. While antibiograms are common in hospitals, they are rarely shared with the wider community.^4^ Many national treatment guidelines for common outpatient infections refer to local resistance patterns for antibiotic selection; however, if antibiograms are unavailable, outpatient prescribers will be left with considerable uncertainty when selecting empiric therapy.^5–8^ Furthermore, in any given city or region, patients are unlikely to use a single health system, limiting a single hospital’s antibiogram predictive abilities.

Prior publications have documented the feasibility of developing cumulative antibiograms at the regional and state levels.^9–11^ These studies recognize the difficulties associated with combining antibiograms from multiple facilities, including formatting issues, adherence to Clinical Laboratory and Standards Institute (CLSI) guidelines, variation in hospital size and case mix, and isolate testing methods.^1,12^ Prior studies combining antibiograms in regions or communities have shown differences from other external-facing surveillance programs such as SENTRY, but have not investigated the clinical utility these tools might have.^11^ We sought to not only create a combined antibiogram for our community but also to find ways to disseminate the data to local providers.

A previous survey showed major gaps in physician knowledge of the antibiogram and even the ability to access an antibiogram.^13^ Subsequently, 73.7% of respondents in this survey desired more education on antibiograms. While prior studies showed availability of antibiograms may impact prescriber decisions on case vignettes, no studies directly link antibiogram knowledge with actual prescribing habits.^14^ Nevertheless, education on local resistance patterns may prevent the selection of unnecessarily broad therapies or ineffective antibiotics. This may be particularly useful when employed in outpatient settings where antibiograms may not be readily available.

The purpose of this study was twofold: to develop a combined citywide antibiogram to be shared among healthcare providers in Des Moines, Iowa (known as AntibiogramDSM) and to demonstrate the utility of an educational session supplemented by this combined antibiogram.

## Methods

### AntibiogramDSM synthesis

To collect data for the initial antibiogram, a survey was conducted with antimicrobial stewardship pharmacists at all four health systems in the Des Moines metro area. The survey included questions on antibiogram data collection methods, microbiology panels used, breakpoints, and adherence to CLSI’s 2014 M39 document.^1^ Survey questions were aimed at confirming similarities in antibiogram reporting methodologies among health systems. The survey was administered in 2018 alongside the first collection of antibiograms from 2017. Three health systems submitted 12-mo antibiograms and one submitted an 18-mo antibiogram (extending to July 2016). These four antibiograms represented eight hospitals, including their emergency departments and one including outpatient clinic data, as each health system created only one antibiogram to represent all its institutions, see Table 1 for participating health-system demographics. Antibiogram data for 2017 were collected by converting health-system antibiograms into tabular format extracting the genus, species, gram stain category, antibiotic tested, number of isolates, susceptibility reported, and health-system identifier.

**Table 1.** Demographics of participating health systems

Health system antibiogram	Number of hospitals represented	Descriptors of each hospital	Average census*	Case mix index*
A	1**	Community hospital, public	96.5	1.5522
B	2	Community/Tertiary care hospital, private	411.7	2.1945 (combined)
		Community hospital, private	57.6	
C	4	Community hospital, private	132	1.5082
		Community hospital, private	61	1.3796
		Community/Tertiary care hospital, private with separatepediatric hospital, private	385 (combined)	1.3796 (combined)
D	1	Government hospital	42	Not available

* Measured in the last month of the study period (January 2022).

** Antibiogram contains cultures from clinics associated with hospital.

To make the data suitable for viewing online, Tableau data visualization software (Tableau Software, Inc; Seattle, WA) was utilized to create a typical grid appearance of an antibiogram. The interface is interactive, showing the number of isolates per cell and the number of hospitals contributing to each cell’s aggregate susceptibility. To estimate an aggregate susceptibility value, the total number of susceptible isolates was estimated for each hospital by multiplying the reported number of isolates by the reported susceptibility percentage on the antibiogram. The total number of estimated susceptible isolates for each hospital was divided by the total number of isolates for a given species to re-calculate an aggregate susceptibility. To align with CSLI’s M39 guidelines, species with fewer than 30 total isolates reported were excluded from presentation on final antibiogram visualization, but included in the data set. This allowed for inclusion in the aggregate susceptibility in the total antibiogram unless the 30 isolate rule was not met. One institution aggregated *Staphylococcus aureus* as a single organism instead of categorizing isolates by methicillin-resistant and methicillin-sensitive per CLSI recommendations.^1^ These data were excluded from the final antibiogram. Cases where all species of a genus were combined on an institution’s antibiogram were denoted using “unspeciated” in the species column and presented separately from other organisms in that genus.

Following the pilot using 2017 data, this process was repeated annually for 2018, 2019, and 2020 data. The antibiogram was published on a website in November 2020 for public use. As each year’s data were added to the antibiogram, users could select which year’s data to view or view all available years of data aggregated. Because one institution created 18-mo antibiograms, 12-mo microbiology reports were provided from that institution to include into the data set. This institution had a complex cephalosporin gram-negative cascading report, which led to the censoring of all except cefazolin from that health system in 2018 only. In 2020, one hospital provided data for only half of 2019 due to a major shift in laboratory software. In 2020 and 2021, one health system did not create antibiograms due to stressors related to the COVID-19 pandemic. In 2021, one institution reported separate urine susceptibility for cefazolin versus enterobacterales due to changes in breakpoints. All susceptible isolates were aggregated as reported by each institution, but a footnote was added to AntibiogramDSM to notify viewers of differing breakpoints.

### Educational session

**Figure 1.**
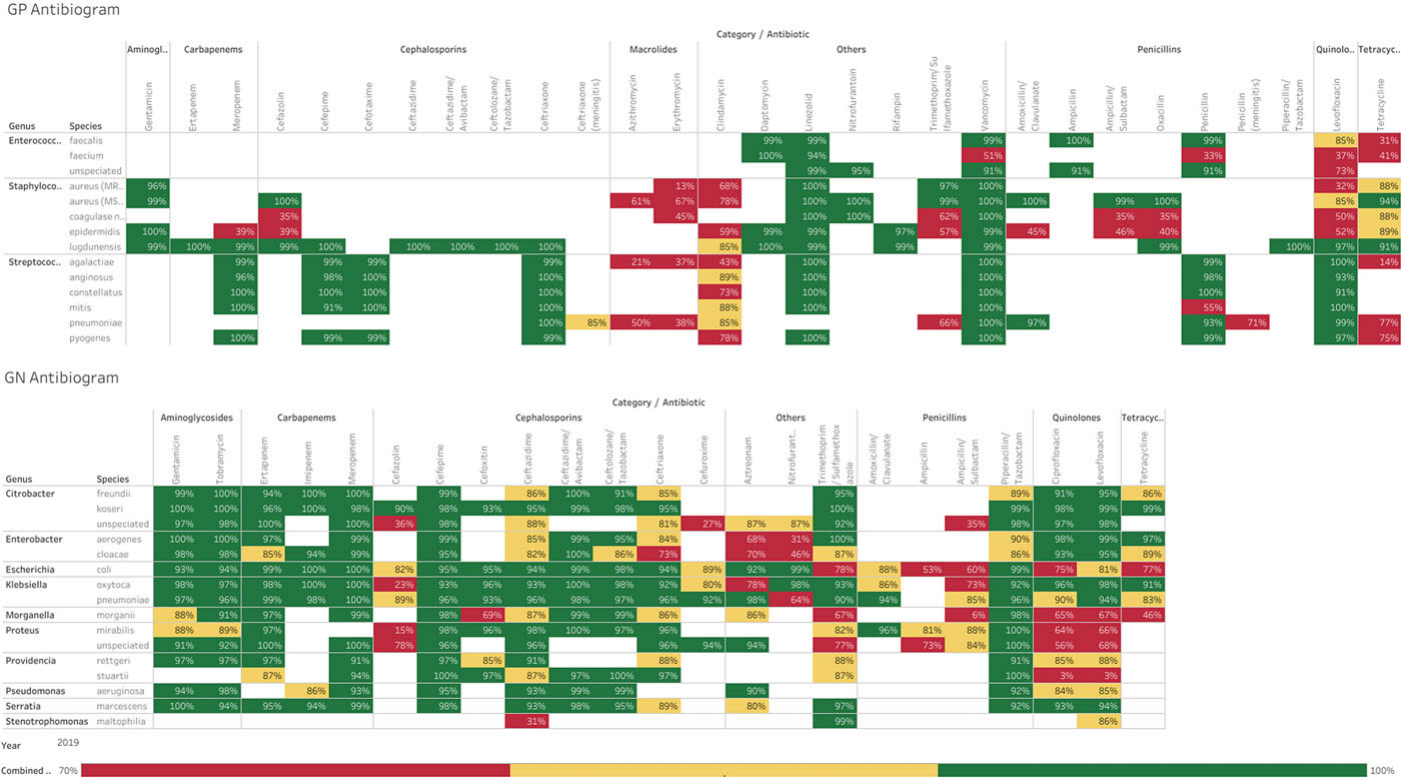
Antibiogram at the time of education session.

Using information from the 2019 antibiogram (see Figure 1), a continuing medical education (CME) module was created by the research team. The CME session was promoted via several professional healthcare societies to outpatient prescribers practicing in central Iowa. Any interested healthcare provider or student was allowed to participate; informed consent was required to access the education surveys. The module was offered live via a web conference once in January 2021, and a recorded version was made available online for on-demand participation for one year. The module included an electronic pre-education survey and assessment, a 60-min presentation, and an electronic post-education survey and assessment. The educational presentation explained how to interpret an antibiogram and reviewed national treatment guidelines for four infectious diseases that rely on local resistance patterns for antibiotic selection: urinary tract infections (UTI), community-acquired pneumonia (CAP), acute bacterial sinusitis (ABS), and diverticulitis.^5–8^ The attendees were encouraged to interact with the AntibiogramDSM website during the session to apply knowledge of local resistance rates to interactive questions. The pre-education survey contained 14 items including demographic information, past or current use of an antibiogram, an estimation of resistance rates of common antibiotics to *Escherichia coli* and *Streptococcus pneumoniae*, estimation of methicillin resistance of *S. aureus* isolates, and four case-based multiple-choice questions on each infectious disease discussed in the presentation. The post-education survey included nine items including four new case-based multiple-choice questions on each infectious disease discussed in the presentation as well as an assessment of the participant’s confidence in addressing the learning objectives. Not all questions were reliant on knowledge of local resistance patterns, but rather the learning objectives of the CME program, most of which were guideline-based recommendations informed by local antibiotic resistance information. Learning objectives and questions can be found in the Supplemental Appendix. Participants were offered CME credit for completing the entire module, but were allowed to skip questions on both the assessment surveys. Personally identifiable information was collected for CME accreditation purposes, but survey responses were de-identified before being sent to the research team.

The proportion of correct responses on the pre- and post-surveys was compared for the UTI, CAP, ABS, and diverticulitis cases as well as mean percentage score on the four cases. McNemar and Welch’s t-tests were used where appropriate. Data were analyzed using SPSS v27 (IBM; Armonk, NY). This study was submitted and exempted from review by the Institutional Review Board at Drake University.

## Results

### AntibiogramDSM 4 yr outcomes

At the time the antibiogram was made available publicly online in November 2020, three annual combined antibiograms had been created using data from 2017 (pilot), 2018, and 2019. At the time the on-demand education session closed in January 2022, four years of data were included, spanning 123,168 isolates with 1,731,569 individual susceptibility results, 57 unique species, and 46 different antimicrobial agents (meningitis breakpoints of ceftriaxone and penicillin were considered as separate entities). In any individual year, the combined antibiogram reported 30–47 unique species of bacteria and their susceptibility to 30–42 different antimicrobial agents (not all species were tested against all agents) (see Table 2).

**Table 2.** Combined antibiogram results after 4 yr

	2017 Combined Pilot Antibiogram	2018 Combined Antibiogram	2019 Combined Antibiogram	2020 Combined Antibiogram
	(4 antibiograms)*	(4 antibiograms)	(3 antibiograms)**	(3 antibiograms)
Isolates (range)	57,116 (964–49,539)	30,521 (530–22,812)	24,896 (2,706–17,453)	10,635 (2,288–4,342)
Total susceptibility Results (range)	895,744 (12,815–814,668)	285,314 (6,046–207,130)	407,823 (28,653–322,974)	142,688 (24,693–65,019)
Gram-positive species (range)	19 (4–17)	22 (4–19)	14 (6–10)	19 (6–15)
Gram-negative species (range)	28 (5–26)	25 (5–23)	16 (7–14)	25 (6–23)
Duplicate species on ≥2 antibiograms	17	17	16	16
Duplicate species on ≥3 antibiograms	12	13	8	7
Duplicate species on 4 antibiograms	6	6	NA	NA
Antibiotics displayed (range)	35 (16–33)	36 (20–34)	35 (20–26)	30 (18–25)
Duplicate antibiotics reported on ≥2 antibiograms	26	29	25	27
Duplicate antibiotics reported on ≥3 antibiograms	20	21	14	14
Duplicate antibiotics reported on 4 antibiograms	15	15	NA	NA

Note. Date represents the year of the data origination, not the year of antibiogram publication. NA, not applicable.

* One health system reported 18 mo of data in the pilot.

** One health system reported only 6 mo of data due to software changes.

In any year, at least 65% of the antibiotics were reported by two or more reporting institutions. Across all institutions, there was reporting on 15 common antimicrobial agents. Species were not as commonly reported across institutions, in part due to specific classifications of certain species, or reporting at the genus level for some bacteria.

### Educational session outcomes

After an year, 45 individuals participated in the educational session (15 during the live web conference, 30 using the recording). The demographic information of participants can be found in Table 3. A majority of participants (57.6%) were prescribers (physicians, physician associates, and nurse practitioners) and 27.6% were students. The majority practiced in the outpatient environment (71.9%). Family medicine was the most common (39.4%) subspecialty represented. The mean years in practice was 14.3 yr among those participants who were not students. Only 17.8% of participants had access to an antibiogram and 11.1% currently used an antibiogram in their practice. Of those currently using an antibiogram, all were using a current antibiogram from a health system which reported into AntibiogramDSM.

**Table 3.** Demographics of educational session participants

**Profession (** ***n*** **= 45 respondents)**	
Physician	35.6% (16)
Students (PA, medical)	26.7% (12)
ARNP	11.1% (5)
Nurse	11.1% (5)
PA	11.1% (5)
Pharmacist	4.4% (2)
**Specialty (** ***n*** **= 33 respondents)**	
Family Medicine	39.4% (13)
Internal Medicine	15.1% (5)
Emergency Medicine	9.1% (3)
Obstetrics/Gynecology	9.1% (3)
Geriatrics	6.1% (2)
Podiatry	6.1% (2)
Pediatrics	6.1% (2)
Public Health	3.0% (1)
Dermatology	3.0% (1)
Administration	3.0% (1)
**Practice Area (** ***n*** **= 32 respondents)**	
Mostly outpatient	71.9% (23)
Mostly inpatient	12.5% (4)
Mix of both	15.6% (5)
**Antibiogram Use (** ***n*** **= 45 respondents)**	
Never used an antibiogram and no current access	44.4% (20)
Previously used an antibiogram, but no current access	37.8% (17)
Currently use an antibiogram	11.1% (5)
Have access to an antibiogram, but do not use it	6.7% (3)

Note. Reported as % (*n*). ARNP, Advanced Registered Nurse Practitioner; PA, Physician Associate.

Prior to education, when asked about local resistance of *E. coli* to various antibiotics, most participants underestimated resistance to quinolones (69.8% underestimated, 30.2% correct), trimethoprim/sulfamethoxazole (69.8% underestimated, 30.2% correct), and third-generation cephalosporins (55.8% underestimated, 44.2% correct). Most overestimated resistance to nitrofurantoin (51.5% overestimated, 48.8% correct). When asked about local *S. pneumoniae* resistance to various antibiotics, most participants overestimated resistance to quinolones (65.9% overestimated, 34.1% correct) and penicillin (71.4% overestimated, 28.6% correct), while most underestimated macrolide resistance (69% underestimated, 31% correct) and correctly identified third generation cephalosporins’ low resistance rates (57.1% correct, 42.9% overestimated). The local *S. aureus* methicillin resistance rate was 37% on the combined antibiogram; 23.7% correctly identified this rate within a ±10% range, but most (65.8%) overestimated this rate.

After education, 82.4% (28/34) could identify the role of an antibiogram correctly. Mean scores on the four case-based questions improved after the education session (48.5% vs 80.9%; *p* < 0.001) and 70.6% (24/34) improved their performance. The proportion of correct answers improved for all four case-based questions, but did not reach statistical significance for the ABS case, see Table 4 for results. Post-education correct responses exceeded 80% on all questions except for the CAP case (58.8%).

**Table 4.** Case-based question results

Case	Pre-Education—Correctly Answered	Post-Education—Correctly Answered	*P*-Value
CAP	31.8% (14/44)	58.8% (20/34)	0.022
UTI	47.7% (21/44)	85.3% (29/34)	<0.001
ABS	75% (33/44)	91.2% (31/34)	0.109
Diverticulitis	43.2% (19/44)	88.2% (30/34)	0.002

After completing the education session and cases, 87.1% (27/31) felt somewhat or very confident in their ability to interpret an antibiogram. Similarly, 90.3% (28/31) felt somewhat or very confident in their ability to apply local resistance rates to a case and select appropriate pharmacotherapy for each of the diseases discussed. Ultimately, 83.3% (25/30) stated that they were likely or very likely to continue to use AntibiogramDSM.

In the time during the educational initiative, there were 1272 visits (97.8 per month) to the antibiogram website from 1037 unique visitors; 93.9% of the visits were from the United States with 51.3% from the central Iowa region. An additional 9.7% could not be isolated to any location.

## Discussion

Our study adds to the published literature on the feasibility of collaboration between multiple health systems to create regional antibiograms. Previous published studies by Guarascio, Butler, and Var included a larger number of institutions in their studies including large academic medical centers.^9–11^ The AntibiogramDSM study included data from four separate health systems (representing 8 hospitals) in the Des Moines metro. One main goal of this project was to increase awareness locally related to bacterial resistance. Among those providers that were surveyed in the educational session, 82.2% did not have access to an antibiogram. Very few of the surveyed providers could appropriately identify local resistance patterns for common bacteria such as *E. coli* and *S. pneumoniae*. In fact, no individual correctly identified the resistance range of all of the antibiotics presented in the survey. Prior to education, there was a tendency to overestimate resistance of *S. pneumoniae* to many antibiotics (except macrolides which was underestimated). This may possibly result in unnecessarily broad-spectrum therapies for respiratory tract infections on an outpatient basis. However, *E. coli* resistance to guideline-recommended agents used for UTIs tended to be underestimated by those surveyed, which could lead to treatment failures and repeat exposures to antibiotics. In fact, a recent cohort study showed nonsusceptible, uncomplicated urinary tract infection isolates resulted in 1.4 more prescriptions per patient, higher probability of subsequent healthcare encounters, and $426 higher all-cause costs.^15^ Furthermore, in a survey, 75% of ambulatory care physicians felt that poor access to antibiograms could possibly impact prescribing and be an important cause of antimicrobial resistance.^16^

Our study was multilayered and included the creation of the antibiogram, education, and public availability of the antibiogram. Previous studies by Guarascio and Butler discuss the creation of the regional antibiograms and the compiled susceptibility results.^10,11^ However, these studies did not elaborate on the implementation or utilization of the regional antibiogram. Prior surveys show that ambulatory care physicians recognize the need for additional education related to their prescribing practices, particularly in the area of antibiotic resistance.^16^ This study utilized a local antibiogram as a tool to support education and allow providers to select agents informed by both national guidelines and local resistance patterns. Participants were able to select more appropriate pharmacotherapy in all four of the case-based questions in our survey following education and viewing the combined antibiogram. This achieved statistical significance in all but the ABS case, which already had high performance in the pre-education survey.

One major concern is the concept of using a mix of cultures from patients from the community presenting to a local hospital and those obtained from within the hospital. While all the health systems in this study built their antibiograms this way, this method may result in inaccurate assumptions about community resistance rates due to inclusion of nosocomial pathogens in the sample, particularly among gram-negative pathogens.^17^ A prior comparison of an outpatient antibiogram and inpatient antibiogram found some statistically significant differences in gram-negative resistance to some antimicrobials versus *E. coli*, *Klebsiella*, and *Pseudomonas*. Most variance was within 10%. However, using data sets that include inpatient cultures may be the only method to have adequate isolate counts for outpatient providers to gain access to an antibiogram in some settings. Creation of an outpatient only combined antibiogram would require all health systems to define cultures as inpatient or outpatient consistently, including those with a community-acquired infection, which was not cultured until the patient was admitted to a hospital. It may also require integration of cultures from clinics in the community, which may be operating independently of local health systems. While a combined antibiogram may theoretically present higher resistance rates than the community, a cursory comparison of the 2019 AntibiogramDSM (educational session edition) to SENTRY Public the same year shows lower MRSA rates (37% vs 41.6%) and lower *E. coli* resistance with ciprofloxacin (25% vs 27.1%) or trimethoprim-sulfamethoxazole (22% vs 32.5%).^18^ Furthermore, the participants in the educational session tended to assume greater resistance rates with common pathogens.

Our study had several other limitations. The methodology for each antibiogram year changed slightly. The initial pilot antibiogram included the largest antibiogram representing 18 mo, then the same institution having some cephalosporins censored for enterobacterales, and another organization did not participate due to COVID. This resulted in significant decreases in total isolates included in the antibiogram per year. Also all health systems used different microbiology testing platforms that were updated during various antibiogram years. This might have included different timing to accept updated breakpoint guidance. In this study, we chose to accept how each institution evaluated the susceptibility breakpoint and applied them to their own antibiograms rather than rely on MIC reporting. Such a method would allow greater uniformity in interpretation, but would be laborious. The health systems also created their individual antibiograms within different time frames varying from every year to every 18 mo. This required coordination with a microbiology lab at one institution and may have resulted in more organisms or antibiotics being included in the data set than if the institution’s official antibiogram was provided.

The live CME was presented in January 2021, and online on-demand education was available during the following year. The timing of the educational opportunities occurred during the COVID-19 pandemic. This resulted in a lower than anticipated participation rate in the study. Several of the education session participants were trainees, which may have impacted preeducation responses. Furthermore, the education assessment relied on immediate post-education assessment as a surrogate for knowledge gained and possible changes in prescribing patterns. Some case-based assessments did not require direct knowledge of local susceptibilities. It was felt that assessment of other learning objectives of the CME program was important to address as well. Regardless, in pre-education, it appeared that there was a knowledge gap in the estimation of local resistance patterns. Availability of local resistance data is crucial to informing the development of infectious disease CME. Future studies should assess if the presence of a combined local antibiogram impacts actual prescribing quality.

Following the completion of this study, CLSI released updated guidance related to the creation of antibiograms.^19^ Included in this document are recommendations for multifacility antibiograms and considerations for collection of data to be included in such antibiograms. Data could be centrally collected by a single laboratory or with isolate-level data to produce an antibiogram similar to methods of a single hospital, but this may be an unrealistic option for competing health systems. Although combining individual antibiograms may lead to some inaccuracies due to variability in development methods, lab abilities, and antibiotics presented, it is a more practical way for separate institutions to merge antibiograms. It requires that each stakeholder communicates changes in their processes, particularly with recent changes in CLSI breakpoints.^20^

Health systems should consider collaborating and creating combined antibiograms to be shared with the community. Such tools can become important pieces of public health infrastructure. Combined local antibiograms expand access to information about local resistance patterns and when paired with education can improve antibiotic selection.

## Supporting information

Miesner et al. supplementary material Miesner et al. supplementary material

## Supplementary material

For supplementary material accompanying this paper visit http://doi.org/10.1017/ash.2023.450. click here to view supplementary material
