# Supplementary material for: AntibiogramDSM: a combined local antibiogram and educational intervention
Source: Antimicrob Steward Healthc Epidemiol. 2023 Oct 23;3(1):e179. doi: 10.1017/ash.2023.450 (PMC10654988; doi:10.1017/ash.2023.450)
Supplement: Miesner et al. supplementary material [file S2732494X23004503sup001.docx]

**Supplemental Appendix – Educational Session Survey and Assessments**

**A. SESSION LEARNING OBJECTIVES**

- Explain how to interpret an antibiogram and its role in empiric antibiotic selection.
- Recall current outpatient treatment recommendations for urinary tract infections, community-acquired pneumonia, sinusitis, and diverticulitis.
- Apply local resistance patterns to a patient case and select appropriate empiric pharmacotherapy

**B. PRE-EDUCATION SURVEY**

*Correct answers to assessment questions are noted in italics where relevant.*

1. I am a(n)

- PA
- ARNP
- Physician
- Other

2. I currently practice

- Mostly Inpatient
- Mostly Outpatient
- Mix of both inpatient and outpatient

3. I have been practicing for ____ years

4. Area of practice (may check multiple areas if needed)

- Allergy/Immunology
- Cardiology
- Dental/oral surgery
- Ear, Nose, and Throat
- Emergency / Urgent Care
- Endocrinology
- Family medicine
- Gastroenterology
- Geriatrics
- Hospice or Palliative Care
- Hospital medicine
- Infectious Disease
- Internal medicine
- Mental Health / Psychiatry
- Nephrology
- Obstetrics/Gynecology
- Orthopedics
- Pediatrics
- Podiatry
- Pulmonary medicine
- Urology
- Surgery
- Other: _______

Antibiograms are usually graphical tables that report susceptibility patterns of organisms versus common antibiotics. These reports are a summary of microbiology reports in an institution over a fixed period of time (usually one year).

5. Tell us about your history of access to Antibiograms

- I have never used an antibiogram before
- I have used an antibiogram in the past, but do not have access to one currently
- I have access to an antibiogram, but do not use it***
- I currently use an antibiogram***

***5a. What institution created the antibiogram you have access to currently?

- _________________________________________
- I don’t know

***5b. What year is the antibiogram you have access to?

- _________________________________________
- I don’t know

6. How would you categorize the resistance of ***Escherichia coli*** to the following antibiotics for patients in central Iowa?

Low (<10%) Moderate (10-20%) High (>20-25%)

Quinolones *X*

Trimethoprim/sulfamethoxazole *X*

Nitrofurantoin *X*

3^rd^ Generation Cephalosporins *X*

7. How would you categorize the resistance of ***Streptococcus pneumoniae*** to the following antibiotics for patients in central Iowa?

Low (<10%) Moderate (10-20%) High (>20-25%)

Quinolones *X*

Penicillin *X*

3^rd^ Generation Cephalosporins *X*

Macrolides *X*

8. What percentage of Staphylococcus Aureus cultured from patients in central Iowa is likely to be methicillin resistant? (i.e. MRSA) ____________ *(37%)*

9. You are treating a 44-year-old male with minimal past medical history for **community-acquired pneumonia** and plan to treat him on an outpatient basis. Assuming no allergies or other contraindications to therapy, **which would be your preferred recommendation?**

- Azithromycin 500mg x1, then 250mg daily
- Levofloxacin 750mg daily
- *Amoxicillin 1gm three times daily*
- Doxycycline 100mg twice daily

10. A 22-year-old female presents to your clinic for burning and pain upon urination and suprapubic pain. She has no systemic signs of infection and you diagnose her with **cystitis** after a positive urine dipstick test. She is not pregnant and has no medication allergies. **What is your preferred antibiotic to suggest in this situation?**

- *Nitrofurantoin*
- Trimethoprim/sulfamethoxazole
- Ciprofloxacin
- Levofloxacin

11. A 7-year-old female presents to your clinic with a fever (39C) and purulent nasal discharge. She started having symptoms of a viral upper respiratory infection about 10 days ago which seemed to have gotten better, but then significantly worse three days ago. Her mother became concerned yesterday as her fever has not gotten better with acetaminophen. You suspect bacterial sinusitis. **What would be the best empiric option for this patient?**

- Amoxicillin 20mg/kg twice daily
- *Amoxicillin/clavulanate 45mg/kg twice daily*
- Azithromycin 10mg/kg daily
- Levofloxacin 10mg/kg daily

12. A 54-year-old male comes to your emergency room with constant left lower quadrant tenderness and diarrhea over the past two days. He is nauseous, but has been able to keep down fluids and bland food. He has a history of hypertension, diverticulosis, and hyperlipidemia. A CT of the abdomen shows left sided diverticulitis without any evidence of abscesses or perforation. His vital signs have been stable and he thinks he could likely recover at home. You suggest a clear liquid diet for the next few days. What else would you suggest in this situation?

- Ciprofloxacin + metronidazole for 7 days
- Amoxicillin/clavulanate for 4 to 7 days
- Trimethoprim/sulfamethoxazole + metronidazole for 10 days
- *No antibiotics at this time; return in 2-3 days for follow up*

**C. POST-EDUCATION ASSESSMENT**

*Correct answers and explanations to assessment questions are noted in italics where relevant.*

1. An antibiogram is best used for:

- Selection of antibiotic after cultures and sensitivities have returned
- Choosing an antibiotic when the diagnosis is uncertain (e.g. fever of unknown origin)
- Guidance of antibiotic dosing
- *Empiric selection of an antibiotic for a known bacterial infection when cultures and sensitivities are not available*

*Feedback provided after assessment submission:*

***ANSWER:*** *Empiric selection of an antibiotic for a known bacterial infection when cultures and sensitivities are not available*

***RATIONALE:*** *Antibiograms have many uses, including providing guidance for empiric antimicrobial therapy where the likely organisms are known. Antibiograms assist healthcare providers with prediction of local resistance based on the most recent cultures (usually over the past year) that have been collected from a population. Antibiograms are not useful once sensitivities have returned; treatment should be tailored to the individual in that situation. Antibiograms are generally not useful when the diagnosis is uncertain because the likely infecting organisms would be uncertain as well. While some susceptibilities reported by labs for some organisms are dose dependent, antibiograms generally do not provide drug dosing information.*

***OBJECTIVE:*** *Explain how to interpret an antibiogram and its role in empiric antibiotic selection.*

2. You are seeing a 65-year-old male with a past medical history of COPD, hypertension, and hyperlipidemia in your clinic. You believe he has **community-acquired pneumonia.** He has a CURB-65 score of 1 and you feel that he could be treated on an outpatient basis. He notes a remote history of itching when receiving penicillin. **Which would be your preferred recommendation?**

- Azithromycin 500mg x1, then 250mg daily
- *Levofloxacin 750mg daily*
- Amoxicillin/clavulanate 875mg twice daily
- Doxycycline 100mg twice daily

*Feedback provided after assessment submission:*

***ANSWER:*** *Levofloxacin 750mg daily*

***RATIONALE:*** *According to the 2019 American Thoracic Society / Infectious Disease Society of America guidelines for Diagnosis and Treatment of Adults with Community-acquired Pneumonia outpatient adults with major comorbidities (chronic heart, lung, liver, or renal disease; diabetes mellitus; alcoholism; malignancy; or asplenia), either a respiratory fluoroquinolone or a selected beta-lactam plus either a macrolide or doxycycline should be used. Given our local resistance patterns to s. pneumoniae, azithromycin and doxycycline monotherapy are not appropriate.*

*Amoxicillin-clavulanate may be reasonable, but only in combination with either a macrolide or doxycycline to provide coverage of atypical bacteria. Streptococcal resistance to levofloxacin is negligible in central Iowa.*

***OBJECTIVE:*** *Recall current outpatient treatment recommendations for urinary tract infections, community-acquired pneumonia, sinusitis, and diverticulitis. Apply local resistance patterns to a patient case and select appropriate empiric pharmacotherapy.*

3. A 52-year-old female presents to your clinic for frequent urination and dysuria. She has felt feverish subjectively, but her temperature and all other vitals were normal today in the clinic. She denies flank pain and has not had any vomiting episodes. A urine dipstick test is positive for leukocyte esterase and nitrites and you send her urine for culture and sensitivities. She has a past medical history significant for hypothyroidism and hypertension. **What do you recommend for empiric therapy in this case?**

- *Nitrofurantoin*
- Trimethoprim/sulfamethoxazole
- Ciprofloxacin
- Doxycycline

*Feedback provided after assessment submission:*

***ANSWER:*** *Nitrofurantoin*

***RATIONALE:*** *According to the 2011 Infectious Disease Society of America and European Society for Microbiology and Infectious Diseases guidelines for treatment of urinary tract infections in women, agents used empirically in the setting of cystitis should have a uropathogen resistance rate of <20% in order to be used routinely. While Trimethoprim/sulfamethoxazole is frequently a first-line agent for cystitis, local resistance patterns of E. coli preclude use without cultures and sensitivities. Similarly, doxycycline has high rates of E. coli resistance, but is not among the agents recommended in guidelines. Not only do fuoroquinolones like ciprofloxacin have a moderate-high rate of e. coli resistance, but also carry a Black Box warning from the FDA advising against their use in cystitis unless no other options are available. Nitrofurantoin is the best option in this circumstance. Rates of E. coli resistance are very low currently.*

***OBJECTIVE:*** *Recall current outpatient treatment recommendations for urinary tract infections, community-acquired pneumonia, sinusitis, and diverticulitis. Apply local resistance patterns to a patient case and select appropriate empiric pharmacotherapy.*

4. A 27-year-old female presents to your clinic with headaches and facial fullness for the last 2 days. She says she gets lots of sinus infections around this time of year and wants to “get on top of this one early” and antibiotics always seem to help. She has no known drug allergies. **What would be the best option for this patient?**

- *Acetaminophen and fluticasone nasal spray*
- Amoxicillin/clavulanate
- Azithromycin
- Levofloxacin

*Feedback provided after assessment submission:*

***ANSWER:*** *Acetaminophen and fluticasone nasal spray*

***RATIONALE:*** *Antibiotics are indicated in bacterial rhinosinusitis when a patient has (a) persistent symptoms lasting 10 or more days, (b) severe symptoms or signs of high fever (39C or 102F) and purulent nasal discharge or facial pain lasting for at least 3–4 consecutive days, or (c) worsening symptoms or signs following a typical viral upper respiratory infection that lasted 5–6 days and were initially improving. Because this patient meets none of these criteria, initial symptomatic management would be preferred over antibiotics. Clinical improvement is seen in as high as 86% of patients that do not receive antibiotics. The American Academy of Otolaryngology recommends deferral of antibiotic treatment for at least 7 days. Macrolides cannot be recommended due to high S. pneumoniae resistance. Fluoroquinolones carry a Black Box warning from the FDA advising against their use in sinusitis unless no other options are available.*

***OBJECTIVE:*** *Recall current outpatient treatment recommendations for urinary tract infections, community-acquired pneumonia, sinusitis, and diverticulitis. Apply local resistance patterns to a patient case and select appropriate empiric pharmacotherapy.*

5. A 50-year-old female comes to your emergency department with left lower quadrant tenderness and diarrhea. She is nauseous and has been able to keep down small amounts of fluids, but has no appetite after a few vomiting episodes after eating. She has a history of diabetes, diverticulosis, hyperlipidemia, and hypertension. She states that she had diverticulitis that “felt just like this” about 3 years ago. In fact a CT shows diverticulitis of the ascending colon without abscess. Her vitals are stable other than a temperature of 38.3C. Her labs are also normal other than a C-reactive protein of 2.4mg/dL. She has a history of mild rash to amoxicillin. She is refusing admission due to concerns about her children at home, but will follow up if needed. What else would you suggest in this situation of moderate severity diverticulitis?

- Ciprofloxacin + clindamycin for 7 days
- *Cefdinir + metronidazole for 7 days*
- Trimethoprim/sulfamethoxazole + metronidazole for 10 days
- Levofloxacin for 14 days

*Feedback provided after assessment submission:*

***ANSWER:*** *cefdinir + metronidazole for 7 days*

***RATIONALE:*** *Patients with mild symptoms of diverticulitis may not necessarily see symptom improvement with antibiotics. In this case, the patient has moderate symptoms due to poor oral tolerability. She should receive antibiotics in the outpatient setting. Infectious Diseases Society of America/Surgical Infection Society recommend that If resistance to a given antibiotic is present in 10%–20% or more of isolates of a common intra-abdominal pathogen in the community, use of that agent should be avoided. As a result, trimethoprim/sulfamethoxazole and fluoroquinolone based regimens are not appropriate in our community. In addition, this patient’s diabetes may be adversely impacted by the dysglycemic effects of fluoroquinolones. Treatment durations for diverticulitis are typically 4-7 days. Cefdinir + metronidazole is the best option.*

***OBJECTIVE:*** *Recall current outpatient treatment recommendations for urinary tract infections, community-acquired pneumonia, sinusitis, and diverticulitis. Apply local resistance patterns to a patient case and select appropriate empiric pharmacotherapy.*

6. Following this educational session, are you more confident in regards to your ability to explain how to interpret an antibiogram and its role in empiric antibiotic selection?

- Very Confident
- Somewhat Confident
- In Between
- Somewhat Unconfident
- Very Unconfident

7. Following this educational session, are you more confident in regards to your ability to recall current outpatient treatment recommendations for urinary tract infections, community-acquired pneumonia, sinusitis, and diverticulitis?

- Very Confident
- Somewhat Confident
- In Between
- Somewhat Unconfident
- Very Unconfident

8. Following this educational session, are you more confident in regards to your ability to apply local resistance patterns to a patient case and select appropriate empiric pharmacotherapy?

- Very Confident
- Somewhat Confident
- In Between
- Somewhat Unconfident
- Very Unconfident

9. How likely are you to continue to use the antibiogram in your practice?

- Very likely
- Likely
- Uncertain
- Unlikely***
- Very Unlikely***

***9a. Why not? ____________

**D. PRE VS POST CASE COMPARISONS**

- Community Acquired Pneumonia: *PRE-EDUCATION #9 vs POST-EDUCATION #2*
- Urinary Tract Infections: *PRE-EDUCATION #10 vs POST-EDUCATION #3*
- Sinusitis: *PRE-EDUCATION #11 vs POST-EDUCATION #4*
- Diverticulitis: *PRE-EDUCATION #12 vs POST-EDUCATION #5*
